# Bacterial ghosts as adjuvants: mechanisms and potential

**DOI:** 10.1186/s13567-017-0442-5

**Published:** 2017-06-24

**Authors:** Irshad A. Hajam, Pervaiz A. Dar, Gayeon Won, John Hwa Lee

**Affiliations:** 1 College of Veterinary Medicine, Chonbuk National University, Iksan, 54596 Republic of Korea; 2 Department of Medicine, College of Medicine and Life Sciences, University of Toledo, Toledo, OH 43614 USA

## Abstract

Bacterial ghosts (BG) are empty cell envelopes derived from Gram-negative bacteria. They contain many innate immunostimulatory agonists, and are potent activators of a broad range of cell types involved in innate and adaptive immunity. Several considerable studies have demonstrated the effectiveness of BG as adjuvants as well as their ability to induce proinflammatory cytokine production by a range of immune and non-immune cell types. These proinflammatory cytokines trigger a generalized recruitment of T and B lymphocytes to lymph nodes that maximize the chances of encounter with their cognate antigen, and subsequent elicitation of potent immune responses. The plasticity of BG has allowed for the generation of envelope-bound foreign antigens in immunologically active forms that have proven to be effective vaccines in animal models. Besides their adjuvant property, BG also effectively deliver DNA-encoded antigens to dendritic cells, thereby leading to high transfection efficiencies, which subsequently result in higher gene expressions and improved immunogenicity of DNA-based vaccines. In this review, we summarize our understanding of BG interactions with the host immune system, their exploitation as an adjuvant and a delivery system, and address important areas of future research interest.

## Introduction

Vaccination remains the most efficacious tool to control infectious diseases. Traditionally, live attenuated and killed microorganisms have been used to induce protective immune response against a disease. The live organisms are usually attenuated either by serial passaging in cell culture or the selective disabling of genes associated with pathogenesis and/or survival of the pathogen [1, 2]. Although live attenuated organisms elicit potent immune responses, killed microorganisms remain widely used vaccines to control infectious diseases because of the inadvertent risk of infection associated with live vaccines [1, 3]. Microorganisms are killed by harsh attenuation procedures such as treatment of the pathogen with binary ethylenimine (BEI) or complete disruption of the pathogenic organism, for instance, BEI inactivated FMDV vaccine or detergent-split flu vaccine [4–6]. Unfortunately, during this inactivation process most of the essential structural and immunogenic components of microorganisms are denatured resulting in impaired function and non-efficient immune responses [3]. Thus, killed vaccines generally induce low cell-mediated immune (CMI) responses and shorter duration of immunity as opposed to live vaccines [6–8]. In accordance with this notion, newer vaccines such as DNA and subunit vaccines have been extensively tried over the last two decades, so far with only limited success [8, 9]. These next generation vaccines, however, are poorly immunogenic in nature as compared to traditional vaccines, and therefore necessitate an appropriate adjuvant in the vaccine formulation. Furthermore, DNA vaccines are not effectively targeted to the antigen presenting cells (APC) and are not presented properly in the context of appropriate danger signals [10, 11]. Therefore, DNA-based vaccines need a better delivery system to reach their full potential. Thus, novel approaches are constantly being investigated to develop potent vaccines that are not only safe but require fewer immunizations and are highly efficient in special populations, including the elderly and immunocompromised individuals, which generally lack effective vaccines. Bacterial ghosts (BG) represent a potential platform which not only acts as potent candidate vaccines but also provide a tool for efficient adjuvant and vaccine delivery systems. This novel approach has produced promising results to curb infectious diseases, tested both in natural hosts as well as in experimental animals (Table 1).

**Table 1:** **Bacterial ghosts as candidate vaccines**

Candidate vaccine	Model/route	Response	References
*A. pleuropneumoniae*	Pig/i.m; oral; i.n	Th1 type immune response, improved protection against lung colonization, vaccine provided protection against carrier state upon homologous aerosol challenge, no clinical side effects	[88, 89]
*Vibrio cholera*	Rabbit/i.g	Potent generation of serum vibriocidal antibodies and cell mediated immune responses, protection against diarrhea and death following intra lumen challenge with cholera sero groups O1 and O139	[72]
*Pasteurella haemolytica* *P. multocida*	Cattle; mice; rabbit/s.c	Humoral response, complete protection against the challenge	[96]
*E. coli* O157:H7 BG	Mice/i.g	Potent Humoral and CMI responses, protection against lethal heterologous challenge	[97]
*Edwardsiella trada*	Fish/i.p	Protection against Edwardseilosis	[98]
*E. coli* O78:K80	1 day old chick/i.m; oral	Protection against colibacillosis	[99]
*Klebsiella pneumonia* Kpn-3	Piglets	Significantly high production of humoral antibody responses, protection against homologous and heterologous strains	[15]
*S.* Enteritidis	Chicken/s.c; i.m	*Salmonella* specific IgG and intestinal secretory IgA levels, CMI responses, lower internal egg contamination and reduced colonization of internal organs after challenge	[17, 100]
*S.* Gallinarum	Chicken/oral; s.c; i.m	Protection against the virulent challenge, systemic and mucosal antibody response, potent CD4 and CD8+ responses	[71, 101]
*S.* Typhimurium	Chicken/i.m	*Salmonella* specific IgG and sIgA antibody responses, reduced internal organ colonization after challenge	[102]
*Brucella suis*	Mice/i.p	Induced pathogen-specific serum IgG antibody response, humoral and CMI responses, protection against challenge	[103]

i.g: intragastrically, i.m: intramuscular, i.n: intranasal, i.p: intraperitoneally, s.c: subcutaneous.

BG are non-living cell envelopes of Gram-negative bacteria produced by the controlled expression of lysis gene *E* of bacteriophage phiX174 [12, 13]. Electron microscopy analyses have revealed that BG maintain cellular morphology similar to native bacteria where entire cell surface structures including outer membrane proteins, adhesins, LPS and the peptidoglycan layer are preserved [14]. In addition, the foreign antigens have been loaded inside the cytoplasmic lumen or expressed both on the surface and in the periplasmic space of BG [14, 15]. These remarkable properties make BG an attractive tool for vaccine development and antigen delivery system for both humans and animals. The presence of LPS in the BG does not limit its use as an adjuvant or candidate vaccine due to minimal toxicity as compared to free LPS [16]. Owing to the particulate nature of BG and the fact that they contain many well-known TLR agonists, BG have the ability to be effectively recognized by APC and to subsequently elicitate potent immune responses against their own envelope structures or ghost-delivered foreign antigens [17–19]. In accordance with this notion, BG have been successfully used as adjuvants and a delivery system for a number of viral and bacterial antigens (Table 2). The present review will discuss BG production strategies, BG interactions with the host immune system and their exploitation as adjuvants, and suggests important areas of future research interests.

**Table 2:** **Bacterial ghosts as effective adjuvants both in vivo and in vitro**

In vivo studies			
BG + Foreign Ag	Model/route	Response	References
*V. cholerae* ghosts — *C. trachomatis* Ags	Mice/i.m	Induced local genital mucosal as well as systemic Th1 responses, adoptive transfer of T cells from immunized mice provide protection against a *C. trachomatis* genital challenge	[72]
*E. coli* ghosts-SbsA/Omp26 fusion proteins	Mice/i.p	Omp26 specific antibody response	[81]
*M. haemolytica* BG- beta-galactosidase	Mice/i.d; i.m	Antigen specific humoral and cell mediated immune response, mixed type Th1/Th2, efficient maturation of DC	[10]
*E. coli* ghosts-hepatitis B virus core 149 antigen	Mice/s.c	HBcAg-149 specific antibody response	[104]
*Helicobacter pylori* BG-Omp18	Mice/oral	Anti-*H. pylori* and Omp18-specific antibody response, significant reduction of gastric *H. pylori* colonization	[105]
*S.*Ty21a BG-HIV-1 gp140 DNA vaccine	Mice/s.c	HIV specific potent mucosal and systemic antibody response	[18]
*E. coli* O157:H7 BGs-Stxs-Stx2Am-Stx1B	Mice/i.g	Specific IgA/IgG antibody response, stronger intimin specific IgA/IgG antibodies, anti-toxin and anti-adhesion immune protection	[19]
*S.* Typhimurium BG-fimbrial antigens of ETEC	Mice/i.m	Humoral and cell mediated immune response	[92]

In vitro studies			
BG	Cell type	Response	
*V. cholerae*	Human THP-1	Rapid uptake of BG, Induction of Th1 cytokines	[106]
*A. pleuropneumoniae*	Porcine APCs	Efficient internalization and processing of BG, BG increased expression of SWC3, MIL-2, MSA3, and CD80/86 molecules, increased proliferation capacity of T cells	[68]
*M. haemolytica*	Murine DCs	Activation and maturation of dendritic cells, induction of proinflammatory cytokines	[10]
*E. coli*	Human keratinocytes	Efficient internalization of BG, release of the pro inflammatory cytokines IL-6 and IL-8, induction of antimicrobial psoriasin	[50]
*E. coli*	Bovine DCs	Efficient maturation of DC, Induction of Th1/Th2 cytokines, increased capacity of T cells to proliferate	[13]

i.d: intradermal, i.g: intragastrically, i.m: intramuscular, i.p: intraperitoneally, s.c: subcutaneous.

## Production of bacterial ghosts

**Figure 1:**
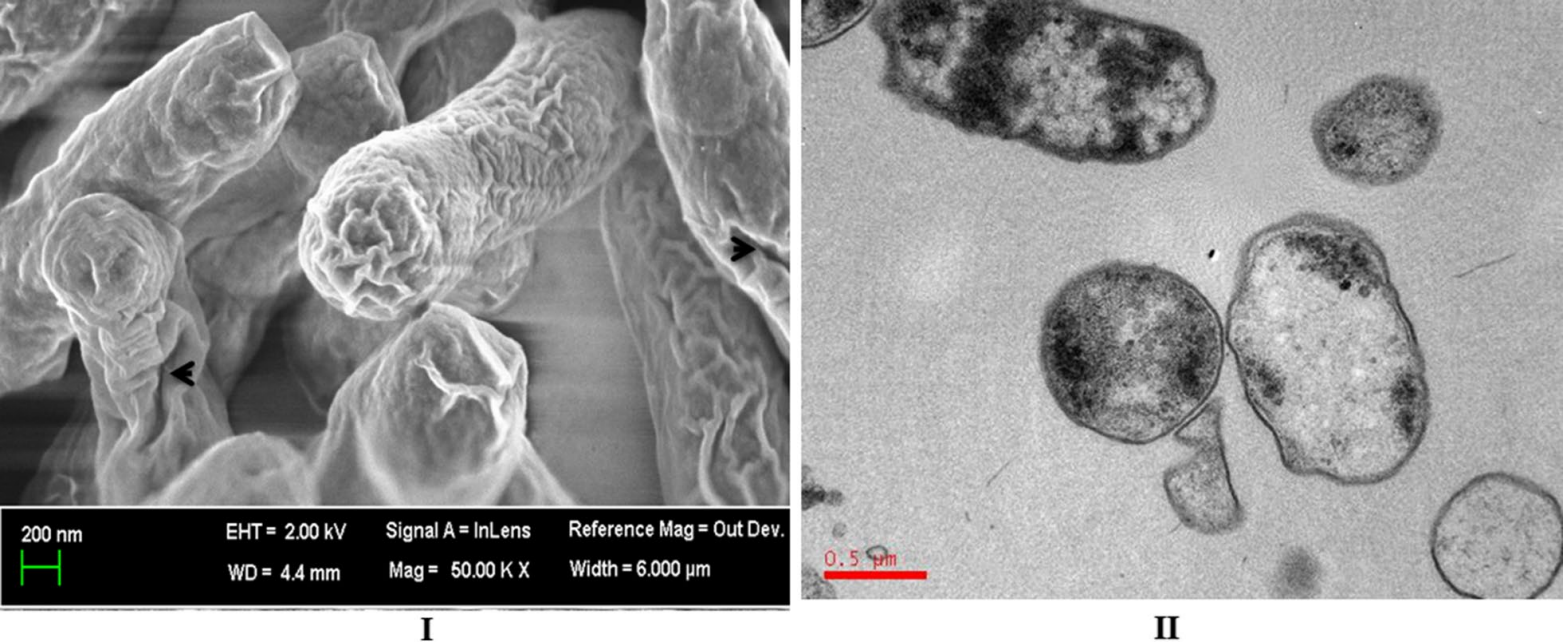
**Scanning (S) and transmission (T) electron microscopies (EM) of BG.** (I) SEM of a BG showing intact cellular morphology except for the presence of a transmembrane tunnel structure as indicated by an arrow. (II) TEM of a BG showing loss of cytoplasmic and nuclear contents.

**Figure 2:**
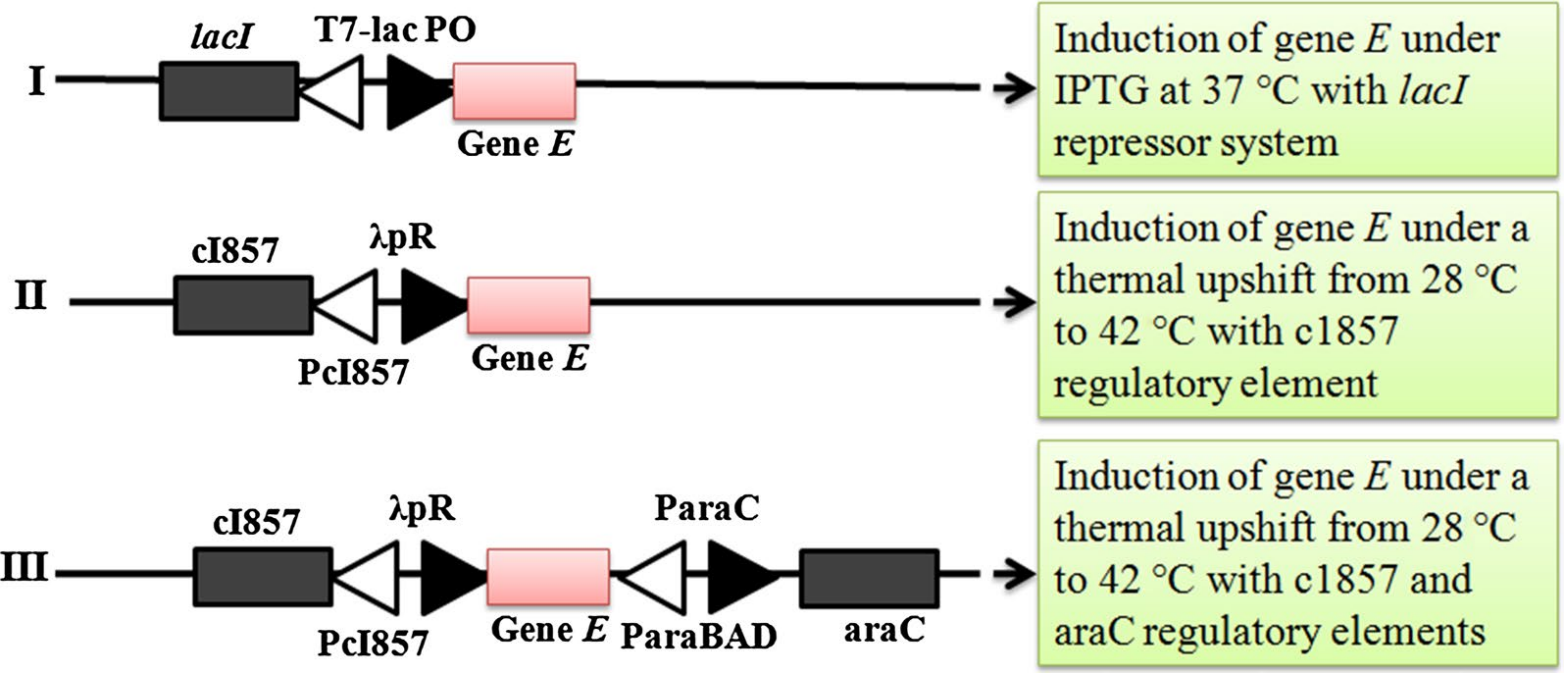
**Expression plasmids used for the synthesis of BG.** (I) Gene *E* expression under the chemical inducer T7-lactose (lac) promoter operator (PO) system with the lac repressor (*lacI*) regulatory element. In this system, bacteria are allowed to grow until 0.3 OD_600nm_ and then gene *E* is induced by the addition of a chemical inducer, IPTG (II) Gene *E* expression under the temperature sensitive lambda promoter (λpR) with the thermo-sensitive repressor c1857 regulatory element. In this system, bacteria are allowed to grow until 0.3 OD_600nm_ and then gene *E* is induced by thermal shift to 42 °C. (III) Gene *E* expression under the λpR with dual c1857 and arabinose-inducible araC protein regulatory elements. The λpR promoter with the thermolabile repressor cI857 suppresses the lysis gene transcription under 28 °C for the normal growth of the bacterial cells. However, the λpR promoter system may be leaky leading to undesired expression of the lysis gene. In this system, the leaky expression of gene *E* at 28 °C is avoided by the anti-sense RNA of the lysis gene produced by the ParaBAD promoter in the presence of L-arabinose that binds to its complementary sense RNA of the lysis gene caused by the leaky λpR promoter.

BG are empty envelopes of Gram-negative bacteria produced by the controlled expression of lysis gene *E* of bacteriophage phiX174 [12, 13, 17, 20]. The role of gene *E* in the lysis of Gram-negative bacteria, *Escherichia coli*, was for the first time reported by Hutchison and Sinsheimer [21], and subsequently this gene was identified by Pollock et al. [22] in heavily UV-irradiated *E. coli* cells. The gene *E* codes for 91 amino acids, possesses lytic action but no inherent enzymatic activity [23, 24]. It is a membrane protein with hydrophobic moieties at its N-terminal region that oligomerizes into a transmembrane tunnel structure [25, 26]. The E-specific tunnel structure spans the inner and outer membrane and is located at the membrane adhesion sites within the host cell [12]. Electron microscopic analysis has revealed that the tunnel formation is associated with the fusion of the inner and outer membrane, sealing the periplasmic space [12]. Due to high osmotic pressure, the cytoplasmic contents including DNA are expelled through the tunnel leaving behind empty cell envelopes known as BG [13, 26] (Figure 1). A study by Witte et al. [27] shows that tunnel formation on the surface of bacteria is not random but occurs at the potential cell division sites, indicating that cell division is mandatory for the formation of BG. The expression of gene *E* can be placed under either transcriptional control of temperature sensitive lambda *pL/pR*-*cI857* promoter or under chemical inducer promoter repressor systems, like *lacPO* or the *tol* expression systems [13, 17, 28, 29] (Figure 2). Studies have shown that expression of gene *E* is sufficient to cause lysis of any Gram-negative bacteria and is much quicker under temperature sensitive systems than the chemical inducer system [13, 20, 30]. We and others have shown that lytic activity of protein E is dependent on the physiological pH (autolytic system) and the growth phase since the non-physiological pH and stationary phase of bacteria have inhibitory effects on the lysis effect [13, 26, 31]. We have generally observed that *E. coli* cultures with OD values above 0.4 show inefficient lysis process while cultures with OD values between 0.2 and 0.3 result in highly efficient BG production [13]. Finally, BG preparation should be free from any viable bacteria and any viable bacteria must be subsequently inactivated. Usually, gene *E* mediated lysis results in complete inactivation in almost all the Gram-negative bacteria except in *E. coli*. We and others have shown that killing in *E. coli* is never absolute and rare detection of non-lysed inactivated cells or reproductive cells are found within the ghost preparation [13, 32, 33]. In order to achieve complete killing, we showed that the addition of H_2_O_2_ to the bacterial culture after 4 h induction of lysis gene *E* causes complete killing of *E. coli* cells and concomitant genomic DNA inactivation [13]. The complete killing and genomic DNA inactivation of *E. coli* can also be achieved by expression of staphylococcal nuclease, SNUC gene, along with the *E*-mediated lysis gene as demonstrated by Haidinger et al. [33]. The SNUC gene is a phosphodiesterase that cleaves single or double stranded DNA or RNA into dinucleotides or nucleosides [33–35], and its action is dependent on Ca++ and Mg++ ions. Alternatively, the viable cells can be lysed by the addition of beta-propiolactone or the addition of either gentamycin or chloramphenicol [15, 36]. However, the expression of the SNUC gene along with gene *E* or the addition of H_2_O_2_ to the culture is advantageous over the use of antibiotics as it completely inactivates any remaining genomic DNA, thus, minimizing the risk of introducing resistance genes or pathogenic islands to resident gut microflora. BG of a number of Gram-negative bacteria have been produced through protein E mediated lysis and subsequently evaluated as candidate vaccines and adjuvants in a number of animal models (Tables 1 and 2).

Besides gene *E* mediated lysis, BG of *E. coli* have also been prepared by the application of high hydrostatic pressure (HP) treatment [37]. In this approach, *E. coli* cells were first sensitized to a high pressure shock through over-expression of *E. coli* K12 Mrr protein. The Mrr protein is a cryptic type IV restriction endonuclease that is activated by mild HP treatment and specifically targets methylated DNA [37]. This study has demonstrated that BG retain their refractility, indicating that they are not lysed or permeabilized unlike ghosts prepared by the protein E mediated lysis. BG derived by this method can be efficiently exploited to deliver subunit or DNA vaccines and do not need to be artificially tethered to the membrane, as is the case with gene *E* mediated BG [38]. Recently, we developed a novel method to prepare BG causing complete killing of the bacteria. In this method, we expressed holin-endolysins along with the gene *E* and observed that the lysis of *Salmonella* bacteria is complete and much faster than the current BG production methods (manuscript submitted). The holin-endolysins are small bacteriophage hydrophobic enzymes and when expressed, form oligomeric pores in the host cell membrane and lysis of the bacteria subsequently [39]. This novel combination of endolysins with the current gene *E* mediated lysis constitutes a safer method to prepare genetically inactivated BG. The BG have also been prepared by the application of minimal concentrations of chemicals including NaOH, SDS and H_2_O_2_, resulting in the production of sponge like structures [40]. This method can be applied to both Gram-negative as well as Gram-positive bacteria; however, the efficacy and potential of these BG to act as adjuvants and a delivery system must be evaluated both in vitro and in vivo. Recently, BG of Gram-positive bacteria *Listeria monocytogenes* have been prepared using a chemical method [41], suggesting that this method could be useful in future vaccine development against important Gram-positive food-borne pathogens. The expression of protein E in Gram-positive bacteria results in cell killing without lysis as the formation of BG depends on the fusion of inner and outer membranes of bacteria, which occurs only in Gram-negative bacteria [12].

## Adjuvant mechanisms of BG

Elicitation of immune response not only depends on the molecular properties of the antigen or on the immunogenic susceptibility of the host but also on the formulation of the antigen. Thus, most vaccine formulations contain immunomodulatory components, broadly termed as adjuvants, to augment the immune responses against the weak immunogenic antigens. Adjuvants mostly potentiate the immunogenicity of vaccine antigens through the stimulation of innate immune receptors present on the cells of the host immune system [6, 42, 43]. The cells of the innate immune system respond to a variety of stimuli including bacterial, viral, parasitic or fungal infections via members of structurally related receptors termed as Toll-like receptors (TLR). TLR are evolutionary conserved type I transmembrane receptors representing a critical link between innate and adaptive immunity. TLR do not possess fine specificity like that of BCR or TCR, the adaptive immune receptors, but individually can respond to a limited but specific number of microbial pathogen associated molecular patterns (PAMP) [42]. The interaction of PAMP with the TLR on the innate immune cells regulates the induction of more efficient adaptive immune responses [44]. TLR sense bacterial cell wall components such as lipopolysaccharide (LPS) (TLR-2/4), lipoteichoic acids (TLR-2/4), CpG DNA (TLR-9), flagellin (TLR-5), and others (reviewed in [45–47]). This sensing initiates an intracellular signaling cascade that culminates in the activation of a variety of pro-inflammatory and immune response genes [44, 47]. The pro-inflammatory cytokines provide augmentary signals, through up-regulation of co-stimulatory and adhesion molecules, essential for the activation of the adaptive immune cells, and in prevention of tolerance to infectious nonself antigens [48]. In recent years, a number of microbial molecules have been used as adjuvants to augment the immune responses of poor immunogenic vaccines. The use of TLR agonists as vaccine adjuvants have shown promising results in animal models and eventually some of them have paved their way into human clinical trials [49].

**Figure 3:**
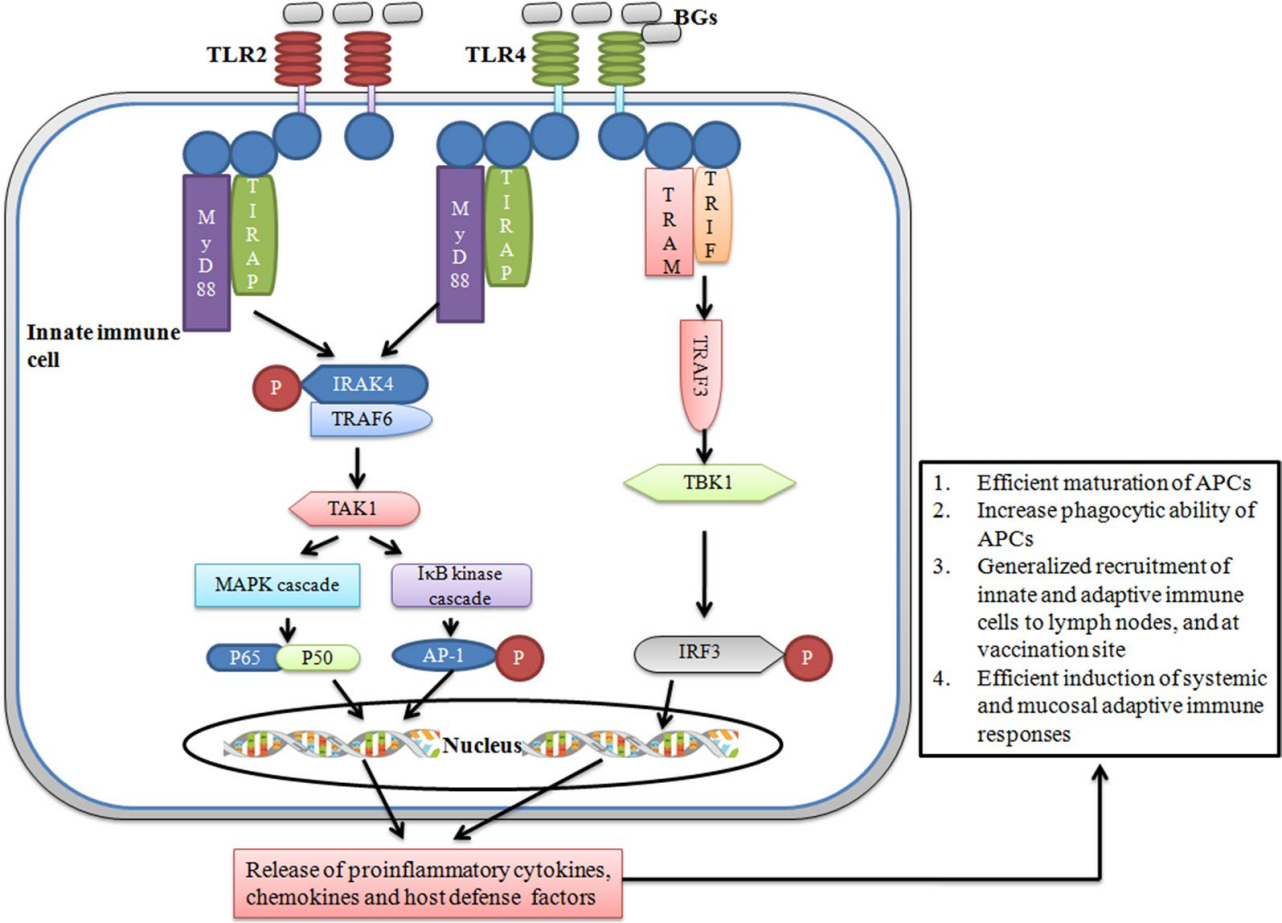
**Signal transduction by BG.** BG activate immune and non-immune cells through TLR2 and TLR4 pathways, culminating in the production of a variety of proinflammatory cytokines, chemokines, and host defence genes via MyD88 and MyD88 independent signaling pathways. Signaling through TLR2 or TLR4 is the MyD88 dependent adapter molecule that passes the signal to MAPK and IkB cascades. These signaling cascades result in the production of NF-kB and AP-1 transcription factors which subsequently induce a variety of genes involved in innate and adaptive immunity. Signaling through TLR4 is also MyD88 independent and occurs via IRF3 pathway, which results in the production of NO and anti-viral cytokines including interferon-α and interferon-β.

**Figure 4:**
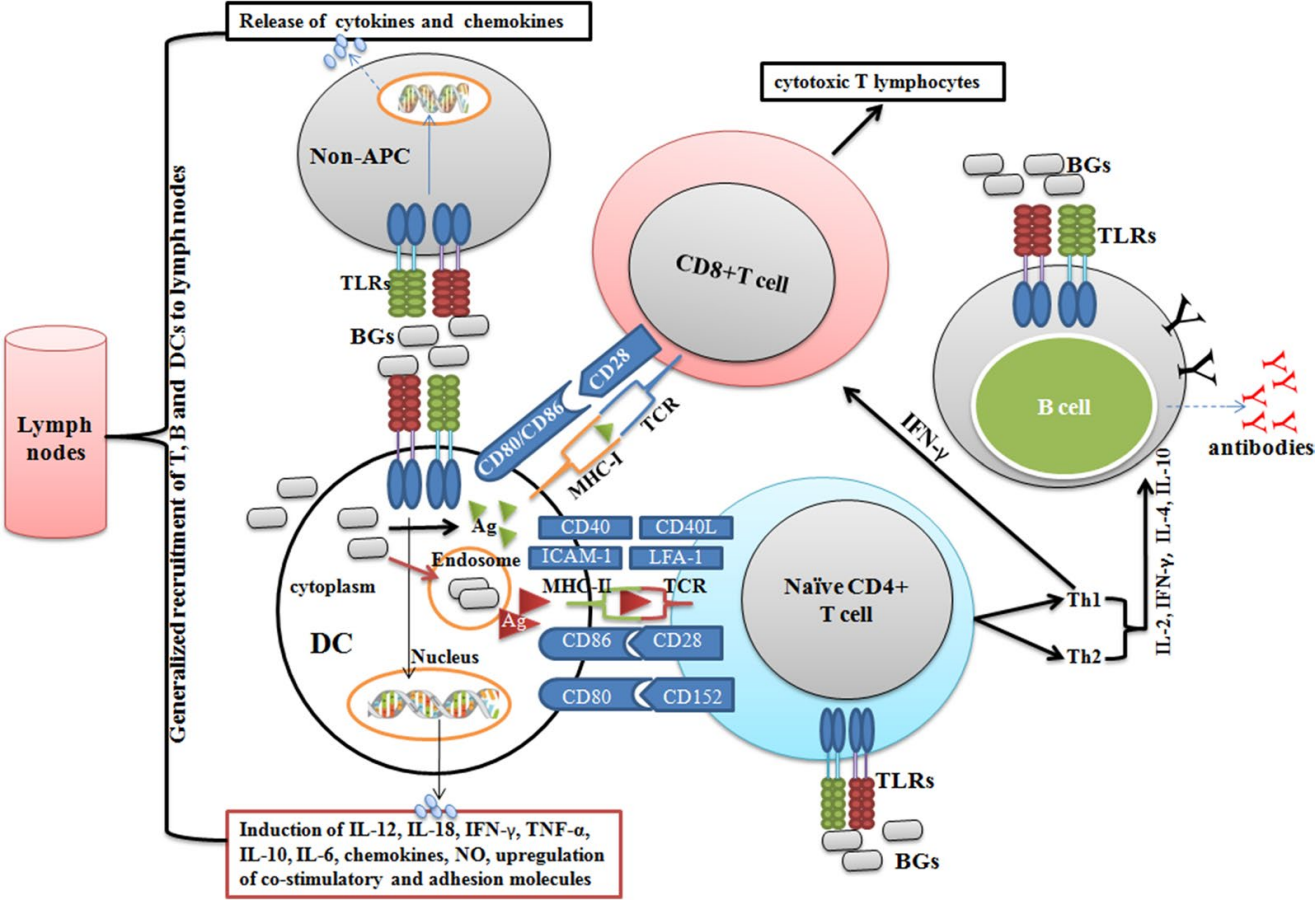
**How BG induce effective humoral and cell mediated immune (CMI) responses.** BG activate immune (DC, macrophages, B and T cells) and non-immune cells (epithelial cells, fibroblasts, and keratinocytes) either through TLR2 or TLR4 pathways. The cumulative effect of the stimulation of these cell types results in the enhanced activation of T and B cells, which subsequently lead to the induction of efficient humoral and CMI responses. The presence of LPS in BG improves the antigen cross presenting ability of DC to CD8+ T cells, and thus helps in the elicitation of potent cytotoxic T cell responses. Moreover, the ability of BG to induce cytokine and chemokine production in a number of lymphoid and non-lymphoid cells results in the generalized recruitment of T, B and DC to lymph nodes that maximize the chances of encounter with their cognate antigen and development of effective immune responses subsequently.

BG contain well-known innate immune stimulating components, and have thus tremendous potential to act as efficient adjuvants. An increasing number of studies have demonstrated that protein E mediated lysis preserves the antigenic nature of BG membrane components including LPS, peptidoglycan or flagella, and are thus identical to the components of native bacteria [14, 15]. Therefore, these envelope structures are efficiently recognized and taken up by immune and non-immune cells [10, 50, 51]. BG mostly stimulate cells through TLR2 and TLR4 pathways [52, 53] (Figure 3), and the presence of multiple TLR on a number of immune and non-immune cells forms the basis of their adjuvant activity. Most of the adjuvants including TLR agonists mediate their activity, in part, by activating the innate immune system including DC activation and maturation, and their recruitment to T cell areas in lymph nodes [54] (Figure 4). DC are unique APC with abilities to prime naïve T cells, and thus play an essential role in the initiation of primary immune responses [55]. They are located at antigen capture sites where they take up antigen and subsequently migrate to lymph nodes for antigen presentation and development of immune responses. BG are efficiently taken up by DC and result in the induction of proinflammatory cytokines, which subsequently upregulate the costimulatory molecules on DC for efficient presentation of foreign antigens to naive T cells [10, 13]. We and others show that BG deliver efficient and early maturation signals to DC, and the induction of Th1 cytokines, especially IL-12, occurs many folds which is the main cytokine driving the stimulation of NK and Th1 cells [10, 13, 56]. The MHCII levels are up-regulated after 12 h exposure to BG [10, 13], indicating that they have the potential to induce early protective immune responses, which are very much required during emergency vaccination. BG also enhance MHC-I expression on DC and the presence of LPS effectively improves the cross presentation and maturation of DC [57, 58]. These findings suggest that BG have the ability to stimulate both humoral and cell mediated immune responses. Besides DC, BG also effectively stimulate monocytes and macrophages and polarize the response toward Th1 [38]. All these factors contribute to the overall potency of BG adjuvated vaccines.

Another factor that contributes to the adjuvant potential of BG is the presence of TLR on non-professional APC. BG are known to stimulate both professional and non-professional APC like conjunctival epithelial cells, fibroblasts, keratinocytes, melanoma cells etc. [50, 51, 59, 60]. These studies have demonstrated that BG are effectively recognized and internalized by non-professional APC, and induce the expression of antimicrobial psoriasin and pro-inflammatory cytokines, IL-6 and IL-8. The cytokine IL-6 helps in the development of effective and potent mucosal immune responses, and protects the host against viral and bacterial infections [61–63]. This indicates that BG have the potential to induce potent mucosal immune responses and could provide non-specific protection against pathogenic organisms, as has been reported with the use of other TLR agonists [64, 65]. The internalization process of BG by non-APC seems to be dependent on the presence of flagellin. A study by Abtin et al. [50] shows that wild type *E. coli* NK9373 BG are efficiently endocytosed by keratinocytes than the mutated aflagellated *E. coli* NK9373 BG. Moreover, the wild type *E. coli* BG resulted in stronger induction of cytokines than the mutated aflagellated BG, suggesting that either TLR5 or inflammasome mediated activation of cells as bacterial flagellin can stimulate either pathway [66]. The elucidation of these activation pathways will result in the better understanding of BG and host cell interactions, and therefore will be helpful for the design of BG-based novel therapies. The presence of TLR on the cells of adaptive immune cells, B and T cells, further contributes to the adjuvant potential of BG. A study by Jalava et al. [67] shows that specific T cell responses were detected after in vitro stimulation of T cells with *Actinobacillus pleuropneumoniae* (APP) ghosts. Felnerova et al. [68] also showed that BG induce a proliferative response in T cells and this proliferation capacity was higher in the cultures including APC than in cultures stimulated with BG only. Thus, BG activate T cells either directly through TLR or indirectly through the presentation of cognate antigen by APC. The presence of LPS is the major contributor factor in the adjuvant potential of BG. A study by Means et al. shows that flagellin-treated DCs have a slightly lower stimulatory T cell effect than LPS-treated DCs, and flagellin stimulation induces the expression of chemokines in DC [69]. The induction of these chemokines is very rapid and results in the recruitment of lymphocytes to the secondary lymphoid sites. This indicates that BG have the potential to recruit not only innate immune cells but also adaptive immune cells at the site of immunization. All these factors lead to the efficient interaction of innate and adaptive immune cells, necessary for the induction of potent immune responses. BG increase the ICAM-1 expression on DC, providing the necessary costimulation for the efficient generation of CD8 + T cell responses [70]. We show that *Salmonella* Gallinarum and *S.* Enteritidis ghosts induce potent CD8+ T cell responses and protect the immunized birds against the lethal challenge [17, 71]. Eko et al. [72] also showed that adoptive transfer of T cells from immunized mice to naive mice provides partial protection against a *Chlamydia trachomatis* genital challenge. These findings, thus, indicate that BG act as efficient stimulators of T cell responses, necessary for the establishment of protective immunity. The effectiveness and adjuvant potential of BG can further be enhanced by expression of potent immunostimulatory molecules on their surfaces. We show that BG carrying *E. coli* heat-labile enterotoxin B subunit induces more potent humoral and cell mediated immune responses than *S*. Enteritidis BG alone [73, 74]. A number of studies have demonstrated that TLR signalling, in particular TLR4, shapes B cell responses including their behavior, migration, and generation of potent antibody formation through class switching [75–77]. This suggests that the presence of LPS in BG might have the ability to stimulate B cells directly through TLR4 pathway and subsequently help in the generation of potent antibody formation, albeit, such studies are completely lacking in the literature. Therefore, a better understanding of BG and B cell interactions would clearly help in the design of effective BG-based novel therapies targeting immune cells, especially tumor immunotherapy, and thus active research is warranted in this regard.

## BG as mucosal vaccines

The BG platform is a novel antigen delivery system endowed with intrinsic adjuvant properties. The native and foreign antigens can be expressed on the surface of ghosts before E-mediated lysis [78], and thus multiple antigens including bacterial, viral, etc. can be presented to the immune system simultaneously. Administration of vaccines through the mucosal route is an attractive idea, albeit, the adjuvants which elicit robust immune responses at mucosal surfaces are lacking. BG have the ability to induce efficient immune responses against envelope-bound foreign antigens, including systemic and mucosal immune responses [19, 38]. The presence of TLR4/TLR5 on epithelial cells, which are often the first and major cell types to encounter infectious and non-infectious agents, form the basis for prospects of BG as mucosal vaccines. The elicitation of immune responses at mucosal surfaces has a potential to eradicate or at least prevent the bad outcome of diseases. During the last two decades, BG against a number of diseases have been tested by mucosal immunization through various routes (Tables 1 and 2). These studies have demonstrated that BG are rapidly taken up by APC and provide complete protection against the lethal challenge. These studies have further demonstrated that BG efficiently activate epithelial cells and culminate in the production of IL-6, NO, chemokines and defensins. These mediators play an important role in activation and recruitment of APC at vaccinal or tissue injury sites, therefore providing protection against the intruding pathogens. We and others showed that a single dose of orally delivered BG provided complete protection against the lethal challenge, and elicited both humoral and cell mediated immune responses [15, 19, 71]. These findings indicate that BG act as potent mucosal candidate vaccines and thus have the ability to overcome the oral tolerance usually associated with orally delivered vaccines, which is a major pitfall for the mucosal route delivery of vaccines [79].

Besides acting as potent mucosal candidate vaccines, BG also act as efficient adjuvants to augment immune responses against the foreign antigens at mucosal surfaces. A study by Szostak et al. shows that the immunization of mice and rabbits with recombinant *E. coli* ghosts carrying various antigens of human immunodeficiency virus (HIV) led to stronger humoral and CMI responses to both the BG components as well as to the viral target proteins [80]. Other studies found that BG induce potent protective mucosal immune responses against the ghost delivered foreign antigens and bias the immune response toward the Th1 type [81, 82]. This indicates that BG act as potent CD8+ T cell mucosal adjuvants, and therefore development of vaccines based on BG might protect humans against a number of intracellular organisms against which conventional vaccines are insufficient or absent. BG also effectively target DNA vaccines to APC at mucosal surfaces. DNA vaccines generally possess low immunogenicity, require high dosage, and are not delivered in the context of an adequate danger signal [11, 83, 84]. Studies have shown that BG act as natural agonists and effectively target DNA vaccines to DC and also increased their transfection efficiencies many folds [10, 18, 78]. These studies have further demonstrated that DNA vaccines delivered through BG have induced more potent immune responses than naked DNA, and biased the immune system toward Th1. The Th1 bias of immune response, indicative of CD8+ T cell activation [85], is important for clearance of persistent infections in natural hosts, and thus BG may act as potential adjuvants for promoting sterile immunity against intracellular pathogens in the susceptible animal species.

## Advantages of BG based vaccines

BG are versatile envelope structures which not only act as potential candidate vaccines but also have the ability to carry envelope-bound antigens in immunologically active forms [17, 19, 73, 78]. BG as candidate vaccines are easy to prepare, have excellent safety profiles, and are stable at room temperature. The conventional methods of inactivation may result in the loss of relevant immunogenic epitopes that are necessary for the efficient stimulation of the immune system [86]. Moreover, the use of whole killed bacteria as a candidate vaccine may result in the introduction of resistance genes or pathogenic islands into host microbes as has been reported by Frosch and Meyer [87]. BG produced by protein E mediated lysis preserves the conformational and non-conformational epitopes, necessary for the proper stimulation of the immune system. BG as vaccines have proven that they are superior to bacterins, and oral or intranasal immunization is superior to parental administration, indicating that they induce more potent mucosal immune responses than killed vaccines [88, 89]. Moreover, the lack of genetic material in the BG vaccine has abolished any hazard of horizontal gene transfer of resistance genes or pathogenic islands to the resident gut flora. Thus, BG constitutes a promising technology for the development of more safe and effective bacterial vaccines. Bacterial infections have always been treated with antibiotic therapies that are often designed to target pathogenic microbes. However, such therapies do not discriminate between the pathogen and the normal microbiota, which is often crucial to keep the body healthy. Continuous treatment of bacterial infections with antibiotics may lead to the development of antibiotic resistance bacterial strains, and thus make treatment regimen ineffective [90]. In accordance with this notion, newer and safer strategies are required to deal with bacterial infections. The BG platform has proven that BG induce potent immune responses against bacterial infections and protect the host against lethal challenge (Table 1). Further, studies have shown that BG have the ability to provide cross protection against heterologous strains and are free from any clinical side effects. This clearly indicates that BG represent the preferred choice over antibiotics to curb bacterial infections.

**Figure 5:**
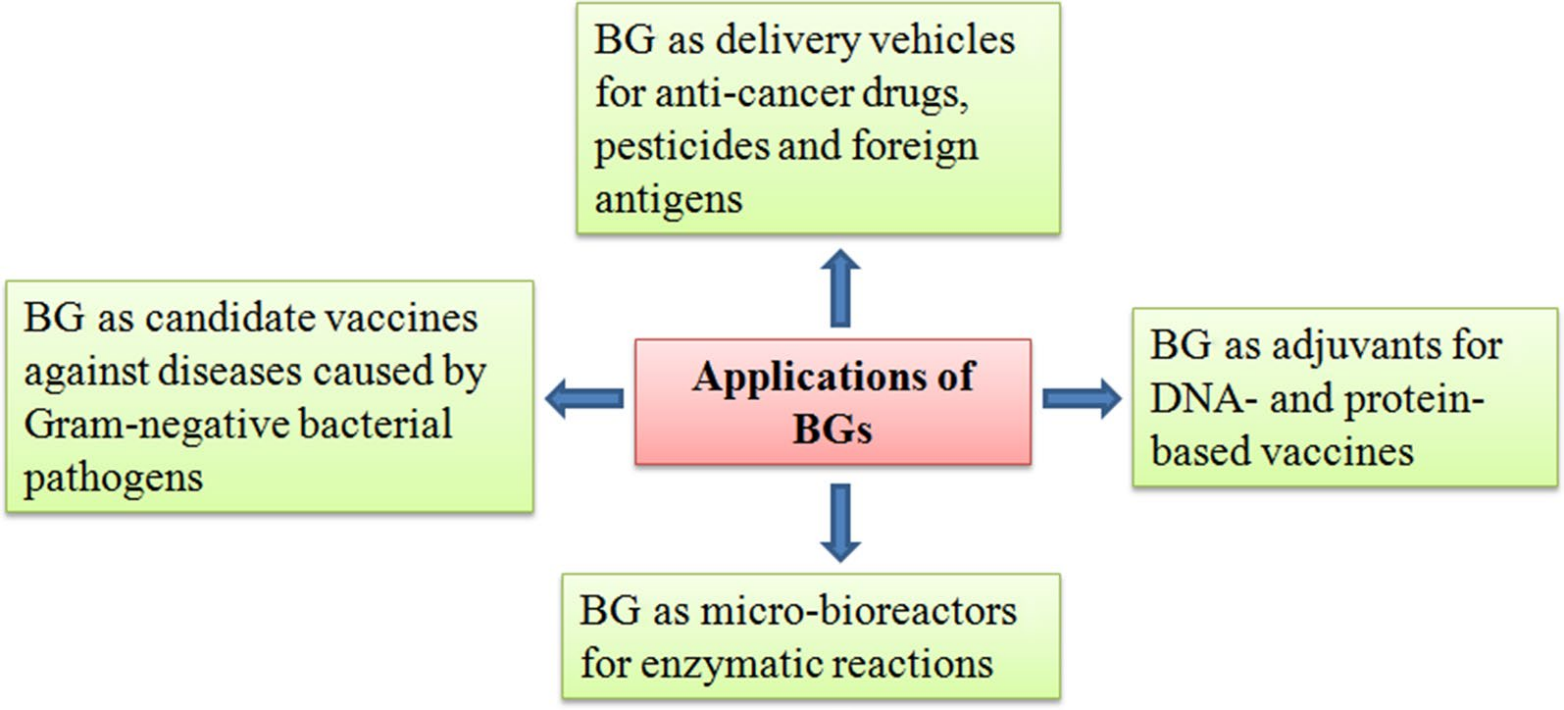
**BG have wide applications both in human and in veterinary fields.**

Besides acting as candidate vaccines, BG have been successfully used as delivery systems for heterologous antigens [10, 18]. BG as delivery systems are safer to conventional viral and bacterial vaccine delivery systems which might revert to their original pathogenic forms. The use of live vectors is usually associated with safety concerns, especially when released under uncontained conditions or when used in immuno-compromised individuals. The advantage of BG over live vectors is their non-living nature. Studies have demonstrated that they are safe even at high doses, and are free of any cytotoxic and genotoxic impact on different types of cells [10, 51, 91]. BG can be exploited to carry foreign antigens in different compartments including outer membrane, periplasmic space and cytoplasm, and thus multivalent vaccines can be designed to induce immunity against multiple infections in a single shot [15, 38]. We and others have shown that BG have great plasticity to create envelope-bound foreign antigens in immunological active form. The expression of envelope-antigen fusion proteins do not interfere with the proper folding and self assembly, and thus preserve the biological activity of proteins as evidenced by the elicitation of potent immune responses against the foreign antigens [17, 18, 78, 92]. BG can also be used as carriers of enzymes, and thus can be exploited to treat patients with defects in metabolism. Studies have shown that they completely preserve the enzymatic activity of enzymes [27, 93, 94], and thus BG can be introduced as novel probiotics by carrying specific enzymes with a certain preference for the gut system. BG can be used in cancer immunotherapy and have the potential to effectively deliver drugs and other biologically active substances to their target sites. Studies have shown that *Mannheimia haemolytica* ghosts effectively targeted hydrophilic cytostatic drug, doxorubicin, to human colorectal adenocarcinoma cells in vitro. This experiment has also shown that the delivery of drugs through BG has enhanced cytotoxic and anti-proliferative activity in the caco-2 cells than using the drug alone [95]. This suggests that BG have the ability to specifically target tumor cells, and thus will allow higher specificity of treatment and a reduction of the total amount of drug per application. All these examples suggest that BG have unlimited potential and benefits (Figure 5). The therapeutic applications of BG are given in Table 3.

**Table 3:** **Therapeutic applications of BG**

Applications	Effects/responses	References
BG as vehicles of anti-cancer drugs	Effective delivery of drugs into cancerous cells, enhanced cytotoxic potential of anti-cancer drugs, suppressed proliferative activities of cancerous cells	[95]
BG as carriers of pesticide drugs	Treatment showed protective and curative effects against plant pathogens, plants showed significant resistance to rainfall	[107]
BG as carriers of immunocontraceptives	Evoked humoral and cell-mediated immune responses against ova proteins, significantly reduced super-ovulation and fertilization	[108]
BG as immunomodulatory agents in cancer immunotherapy	Significant increase in survival rate, significant increase in circulating CD8a+ T cells, significant decrease in metastasis foci area and incidence	[109]
BG as carriers of foreign antigens to treat infectious diseases	Induced mucosal as well as systemic humoral and CMI responses, protection against infectious diseases	[38, 78, 110]
BG as carriers of enzymes, antibiotics and vitamins	Protection of the encapsulated substance against premature degradation and immunological reaction, sustained release of the drug, preservation of enzymatic activity	[38]
BG as carriers of DNA vaccines	Increased DNA transfection efficiencies, increased immunogenicity of DNA-based vaccines, enhanced protective efficacy of DNA vaccines	[10, 38]

## Conclusion and future prospectus

BG are potential envelope structures which not only act as potent candidate vaccines but also have efficient adjuvant and delivery system properties. The future of BG seems to be promising and several considerable studies have reported the effectiveness of BG for the delivery of biotherapeutics, drugs, and vaccines in animal models; however, the future of BG as drug delivery vehicles lie on their ability to effectively deliver biotherapeutics to their target sites. Moreover, a series of extensive and systematic studies are required to implement the BG system in humans. The intrinsic adjuvant properties and the preservation of native envelope structures in BG would definitely replace the use of live or attenuated bacteria as vaccines, which are usually associated with inadvertent risk of infection. Relevant to the use of BG as adjuvants and delivery system, there are many areas worthy of continued investigation. What are the long term consequences of BG in the context of dosage and route of administration? Are other pathways, besides TLR, involved in the recognition of BG? To what extent do the direct effects of BG on T and B cells contribute to the overall adaptive immune response? A better understanding of how BG interact with adaptive immune cells including T, B and Tregs will eventually allow them to be selected for specific vaccines in a targeted and rational manner.

Since BG mediate active immunization against their own envelope components, it would be interesting to elucidate the effect of pre-existing BG-specific immunity on the delivery of heterologous foreign antigens and drugs. BG induce potent proinflammatory cytokine responses in immune cells and therefore, they may not be safe for immunocompromised hosts. Thus, strategies should be devised to minimize their antigenicity so that they can be effectively exploited as adjuvants and delivery systems in immunocompromized hosts. Recently, *E. coli* Nissle 1917 was used as a treatment for inflammatory bowel disease, prevention of allergies, and as a treatment for severe diarrhea in infants and toddlers [51]. The elucidation and role of *E. coli* Nissle 1917 BG in the prevention of these diseases will clearly help in the design of BG-based novel therapies to treat allergic and autoimmune diseases.

## Abbreviations

AP-1: activator protein 1
APCs: antigen presenting cells
BG: bacterial ghost
CMI: cell mediated immune response
DC: dendritic cell
HIV: human immunodeficiency virus
IRAK: IL-1 receptor associated kinase
IRF-3: interferon response factor 3
MAPK: mitogen activated protein kinase
MyD88: myeloid differentiation factor 88
PAMP: pathogen associated molecular patterns
TAK1: transforming growth factor-beta-activated kinase 1
TBK1: TANK-binding-kinase-1
TLR: toll like receptor
TRAF: TNF receptor associated factor
TRIF: TIR domain containing adapter inducing interferon-β
